# Functional connectivity dysfunction of insular subdivisions in cognitive impairment after acute mild traumatic brain injury

**DOI:** 10.1007/s11682-020-00288-5

**Published:** 2020-04-17

**Authors:** Liyan Lu, Fengfang Li, Huiyou Chen, Peng Wang, Hong Zhang, Yu-Chen Chen, Xindao Yin

**Affiliations:** 1 Department of Radiology, Nanjing First Hospital, Nanjing Medical University, No.68, Changle Road, 210006 Nanjing, China; 2 Department of Radiology, The Affiliated Jiangning Hospital of Nanjing Medical University, Nanjing, China

**Keywords:** Mild traumatic brain injury, Cognitive impairment, Functional magnetic resonance imaging, Insula subdivision

## Abstract

**Purpose:**

This study aimed to investigate the early functional connectivity alterations between insula subdivisions and other cortical regions in patients with acute mild traumatic brain injury (mTBI) and subsequently to explore the relationship between functional connectivity changes of insula subdivisions with other cortical regions and cognitive function.

**Methods:**

Fifty-three mTBI patients and 37 age-, gender- and education level- matched healthy controls were included in this study. All participants obtained resting state functional magnetic resonance imaging (rs-fMRI) and clinical and neuropsychological evaluations (Montreal cognitive assessment, MoCA) at the acute stage. Functional connectivity alterations of insula subdivisions and correlations with MoCA were further explored by seed-voxel functional connectivity.

**Results:**

Compared with healthy controls, patients with acute mTBI showed significantly decreased functional connectivity between the L-vAI and the left middle temporal gyrus and right superior frontal gyrus and significantly decreased functional connectivity between the R-vAI and the right middle frontal gyrus and right hippocampus. While significantly decreased functional connectivity were observed between the L-dAI and the right superior frontal gyrus. In addition, significantly increased functional connectivity was observed between the R-PI and the left inferior frontal gyrus. Furthermore, the mTBI group demonstrated positive correlations between performances in orientation and insula and middle temporal gyrus and superior frontal gyrus and middle frontal gyrus functional connectivities. Abstraction scores for mTBI patients positively correlated with functional connectivity between insula and middle frontal gyrus.

**Conclusions:**

The present study demonstrated functional connectivity dysfunction of insula subdivisions and correlations between these alterations and cognitive performance, which provide a novel insight into the neurophysiological mechanism of cognitive impairment in patients with mTBI at the acute stage.

## Introduction

Mild traumatic brain injury (mTBI) accounts for at least 75% of traumatic brain injury (Gardner and Yaffe 2015). Moreover, a proportion of mTBI patients frequently develop cognitive deficitis following acute mTBI and may persist for months and even years after the initial injury, thus imposes an excessive societal burden (Stenberg et al. 2019). However, the underlying pathophysiology of cognitive disorders remains controversial, partly because the cognitive symptoms are not specific because these patients often perform normal neuropsychological test and lack of structural brain damage on conventional anatomical brain computer tomography (CT) and magnetic resonance imaging (MRI)(Scheibel 2017). Therefore, research on pathophysiological mechanisms of cognitive impairment may help to provide strategy for the early diagnosis and treatment of cognitive deficit following acute mTBI.

Insular cortex, traditionally is considered to be responsible for integrating internal and external processes (Tops and Boksem 2011; Uddin et al. 2014). However, growing findings showed that the insula plays a crucial role in response for salience events and mediates the control of cognition (Uddin et al. 2014). Our previous work observed that the gray matter volume was significantly reduced in the insula in acute mTBI patients and the functional connectivity of insula with other brain regions was impaired in mTBI patients during the acute stage (Li et al. 2019; Lu et al. 2019). Following mTBI, previous neuroimaging studies have shown structural and functional connectivity abnormalities in the insula, such as smaller volume, decreased regional homogeneity (ReHo) and cerebral blood flow (CBF), and these alterations were related to cognitive scores. Meanwhile, a large body of literature indicated that insula, as a key node of SN salience network (SN) for initiating network switching, could mediate other important intrinsic connectivity networks (ICNs) such as the default mode network (DMN) and central executive network (CEN) (Seeley et al. 2007). For mTBI patients, abnormal insular connectivity within the SN and dysfunctional interactions with DMN and CEN, and aberrant functional connectivity correlated with neurocognitive functioning were shown by several studies (Sours et al. 2013; Vakhtin et al. 2013; Chand et al. 2017).

Furthermore, parcellation studies using resting state fMRI (rs-fMRI) data revealed that the human insula can be subdivided into the anterior insula (a.i.) and the posterior insula (PI) which are involved in a wide range of cognitive processes, especially in restoring the cognitive functions (Iaria et al. 2008; Taylor et al. 2009; Lu et al. 2019). Recent evidence consistently highlighted that a.i. is in response for cognitive function (Taylor et al. 2009; Chand et al. 2017; Peng et al. 2018; Lu et al. 2019). Additionally, we have previously shown decreased grey matter volumes of a.i. and disrupted functional connectivity of a.i. with other brain regions in mTBI patients during the acute stage (Li et al. 2019; Lu et al. 2019). All these results indicated the vital role of a.i. in the mechanisms of cognitive impairment. Mover, a.i. primarily comprises of the ventral anterior insula (vAI) and dorsal anterior insula (dAI), both of them are associated with arousal/interoceptive awareness, cognitive emotional processing, the dorsal anterior portion is specifically more involved in high level cognitive processes (Peng et al. 2018). Meanwhile, the posterior insular (PI) is a cortical region related to processing of multimodal information such as interoceptive/exteroceptive information and sensory information (Peng et al. 2018). Thus, it is necessary to explore the functional connectivity between the insula subdivisions and other brain regions to explain the cognitive symptoms of mTBI. However, to the best of our knowledge, whether there exist changes of functional connectivity between insula subdivisions and other cortical regions and whether the altered functional connectivity contributed to the pathophysiology of cognitive impairment have not been studied in acute mTBI patients. It is still mixed and not clear whether or how insula subdivisions alterations in patients with cognitive impairment following acute mTBI. To address these issues, we selected the bilateral insula as seed for analysis.

Therefore, the purpose of this study was to examine the abnormalities of functional connectivity between insula subdivisions and other cortical regions at the acute stage and subsequently to explore the association between functional connectivity changes of insula subdivisions and the cognitive test in these patients compared to healthy controls. We hypothesized that (1) patients following acute mTBI have disrupted functional connectivity between insula subdivisions and other cortical regions (2) these alterations would be associated with changes in neuropsychological assessment of cognitive functioning.

## Materials and methods

### Participants

This study was approved by the Institutional Review Board of Nanjing Medical University. All participants provided written informed consent before undergoing MR imaging. Between December 2017 and May 2019, patients with a diagnosis of mTBI within 2 weeks after trauma were prospectively enrolled in this study. mTBI was defined based on the American Congress of Rehabitation Medicine. Inclusion criteria were as follows: (a) patients aged 18 or older; (b) loss of consciousness < 30 min, Glasgow Coma Score (GCS) of 13–15 and post-traumatic amnesia  < 24 h. Exclusion criteria were: (a) previous head injury; (b) history of pre-existing neurological or psychiatric disease; (c) history of illicit drug use or substance abuse; (d) dental appliances that might distort the functional MR images; (e) left-handed. The healthy control participants were recruited through local advertisements who met the same exclusion criteria applied to the patient group.

### Cognitive assessment

Given the emergency care setting, it was not feasible to perform a full battery of neuropsychological assessment. Therefore, a short instrument called the Montreal Cognitive Assessment (MoCA) was used to assess the patients’ neurocognitive status (de Guise et al. 2014). The MoCA is a sensitive cognitive screening test following mTBI and it only requires limited training to administer. The MoCA assesses eight cognitive domains including visuospatial/executive, naming, attention, language, abstraction, memory, and orientation. This test is administered in about 10 min and is scored on a maximum of 30 points. More than 26 was regarded as normal value with a lower score indicating greater cognitive deficit (Wang et al. 2019). All participants completed the MoCA test within 12 h of MRI examination.

### Imaging methods

A 3.0 T magnetic resonance imaging scanner (Ingenia, Philips Medical Systems, Netherlands) with an 8-channel head coil was used for this study and the parallel imaging was employed. Functional images were obtained axially using a gradient echo-planar imaging sequence as follows: repetition time (TR) = 2000 ms; echo time (TE) = 30 ms; slices = 36; thickness = 4 mm; gap = 0 mm; field of view (FOV) = 240 mm × 240 mm; acquisition matrix = 64 × 64; and flip angle (FA) = 90°. The fMRI sequence took 8 min and 8 s. Three-dimensional turbo fast-echo (3D-TFE) T1WI sequence with high resolution: TR = 8.1 mm; TE = 3.7 ms; slices = 170; thickness = 1 mm; gap = 0 mm; FA = 8°; acquisition matrix = 256 × 256; FOV = 256 mm × 256 mm; Fluid-attenuated inversion recovery(FLAIR):TR = 7000 ms; TE = 120 ms; slices = 18; slice thickness = 6 mm; gap = 1.3 mm; FA = 110°; Voxel size = 0.65 × 0.95 × 6 mm^3^. SWI: TR = 22 mm; TE = 34 ms; FA = 20; matrix = 276 × 319; slice thickness = 1 mm; FOV = 220 mm × 220 mm. SWI used 3D gradient echo (GRE) sequence. Diffusional tensor imaging (DTI): TR = 3000 mm; TE = 100; slice thickness = 2.5 mm; gap = 0; b-values = 0 and 1000s/mm^2^; diffusion gradient directions = 32; matrix = 128 × 128; FOV = 256 mm × 256 mm.

### Image processing

Data Processing & Analysis for Resting-State Brain Imaging (DPABI_V2.3_170105) with the following stages was applied for data analysis (Yan et al. 2016). The first 10 volumes were discarded and the remaining 230 consecutive volumes were used for data analysis. Afterwards, slice-timing adjustment and realignment for head motion correction were performed. mTBI and healthy control participant who had a head motion greater than 3.0 mm or a rotation in the x, y, or z directions higher than 3.0◦ were excluded. Data were spatial normalized to the Montreal Neurological Institute (MNI) template (resampling voxel size = 3 × 3 × 3 mm^3^) and smoothed with a Gaussian kernel of 6 mm full width at half maximum (FWHM) to increase signal-to-noise ratio.

**Fig. 1:**
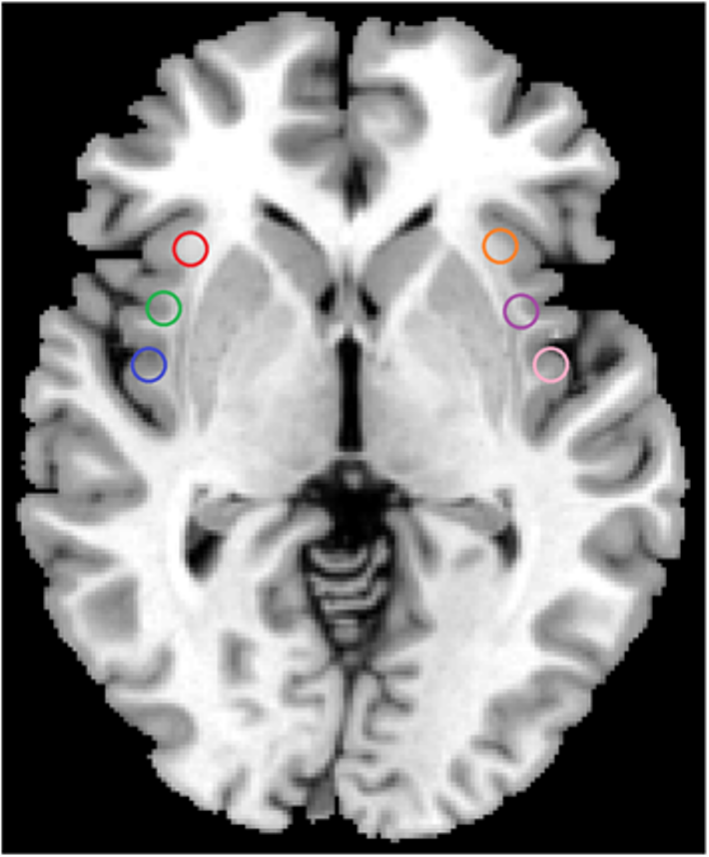
The sub-regions of insula were shown, including: left ventral anterior insula (L-vAI, red), right ventral anterior insula (R-vAI, orange), left dorsal anterior insula (L-dAI, green), right dorsal anterior insula (R-dAI, purple), left posterior insula (L-PI, blue) and right posterior insula (R-PI, pink)

Functional connectivity was analyzed using the REST software. To examine functional connectivity for sub-regions of insula, six spherical 6 mm radius seeds were defined centered on the coordinates for each ROI in Montreal Neurological Institute (MNI 152) space which was showed on Fig. 1, including: left ventral anterior insula (L-vAI, red, MNI = − 33, 13, − 7), right ventral anterior insula (R-vAI, orange, MNI = 32, 10, − 6), left dorsal anterior insula (L-dAI, green, MNI = − 38, 6, 2), right dorsal anterior insula (R-dAI, purple, MNI = 35, 7, 3), left posterior insula (L-PI, blue, MNI = − 38, − 6, 5) and right posterior insula (R-PI, pink, MNI = 35, − 11, 6)(Peng et al. 2018). These coordinates of insular sub-regions seeds were reported previously(Deen et al. 2011). The mean time series of each ROI was acquired for reference time course. Then, Pearson’s correlation coefficients were computed between the mean signal change of each ROI and the time series of each voxel. Functional connectivity was computed between each insular subregion seed and all vertices on the whole brain surface space for each participant. The Fisher’s r-to-z transformed correlation map was then averaged within each group for each seed region to generate the mean functional connectivity distribution (Peng et al. 2018).

### Statistical analysis

To investigate the abnormal functional connectivity between the patients with acute mild traumatic brain injury and healthy controls, two-sample t-test for each seed region was estimated. Then, surface-based cluster-wise correction for multiple comparisons was performed at the significance threshold of p < 0.001 and the cluster size threshold of 13 mm^2^, which was determined by Monte Carlo. For a follow-up analysis, we investigated the relationship between fMRI data and MoCA scores in acute mTBI patients. Pearson’s correlation analyses were performed in a voxel-wise manner using REST software. The statistical threshold was set at corrected p < 0.001 using the same parameters as the group comparison analysis.

Differences in demographic data between mTBI patients and healthy controls were analyzed using between-group t-test for means and two-test for proportions. P < 0.05 was considered to be statistically significant, corrected for age, sex and years of education. Bonferroni correction was used for multiple comparisons in the correlation analyses.

## Results

During the study period, 78 patients with the diagnosis of mTBI after head injury (range, 0–10 days, average, 2.95 days) were recruited. Among these patients, 25 participants were excluded due to pre-existing neurological or psychiatric disease (n = 05), previous head injury (n = 02), dental appliance (n = 05), image artifact (n = 04), or excess head movement (n = 09). The remaining 53 patients were finally analyzed. 37 age, gender and education level matched healthy control participants were also recruited in this study. No significant difference existed in age (P = 0.149), gender (P = 0.138), education level (P = 0.098) and GCS score for both groups. Table 1 is a summary of the basic demographic characteristics of mTBI group and healthy control group. No visible traumatic brain lesions were seen on conventional imaging such as T2 or susceptibility weighted imaging (SWI).

**Table 1:** Demographic characteristics and cognitive performance in patients with mTBI and healthy controls

Characteristics	mTBI(n = 53)	Control(n = 37)	*P* Value
Age (y)	37.96 ± 10.708	41.41 ± 11.243	0.149
Gender(male/female)	27/26	13/24	0.138
Education (y)	12.81 ± 3.058	13.59 ± 3.122	0.098
GCS Score	15	15	
Time since injury(d)	2.95 ± 1.611	--	
MoCA Score	25.15 ± 2.214	26.03 ± 2.154	0.064
Visuospatial/executive	3.89 ± 0.847	4.11 ± 1.048	0.291
Naming	2.94 ± 0.233	2.81 ± 0.397	0.060
Attention	5.62 ± 0.627	5.76 ± 0.495	0.281
Language	2.38 ± 0.657	2.49 ± 0.559	0.413
Abstraction	1.77 ± 0.423	1.95 ± 0.229	0.026^*^
Memory	2.64 ± 1.317	2.81 ± 1.543	0.578
Orientation	5.83 ± 0.379	5.92 ± 0.277	0.228

Data are the mean ± standard deviation; ^*^p < 0.05. mTBI, mild traumatic brain injury; GCS, Glasgow Coma Scale; MoCA, Montreal Cognitive Assessment

**Fig. 2:**
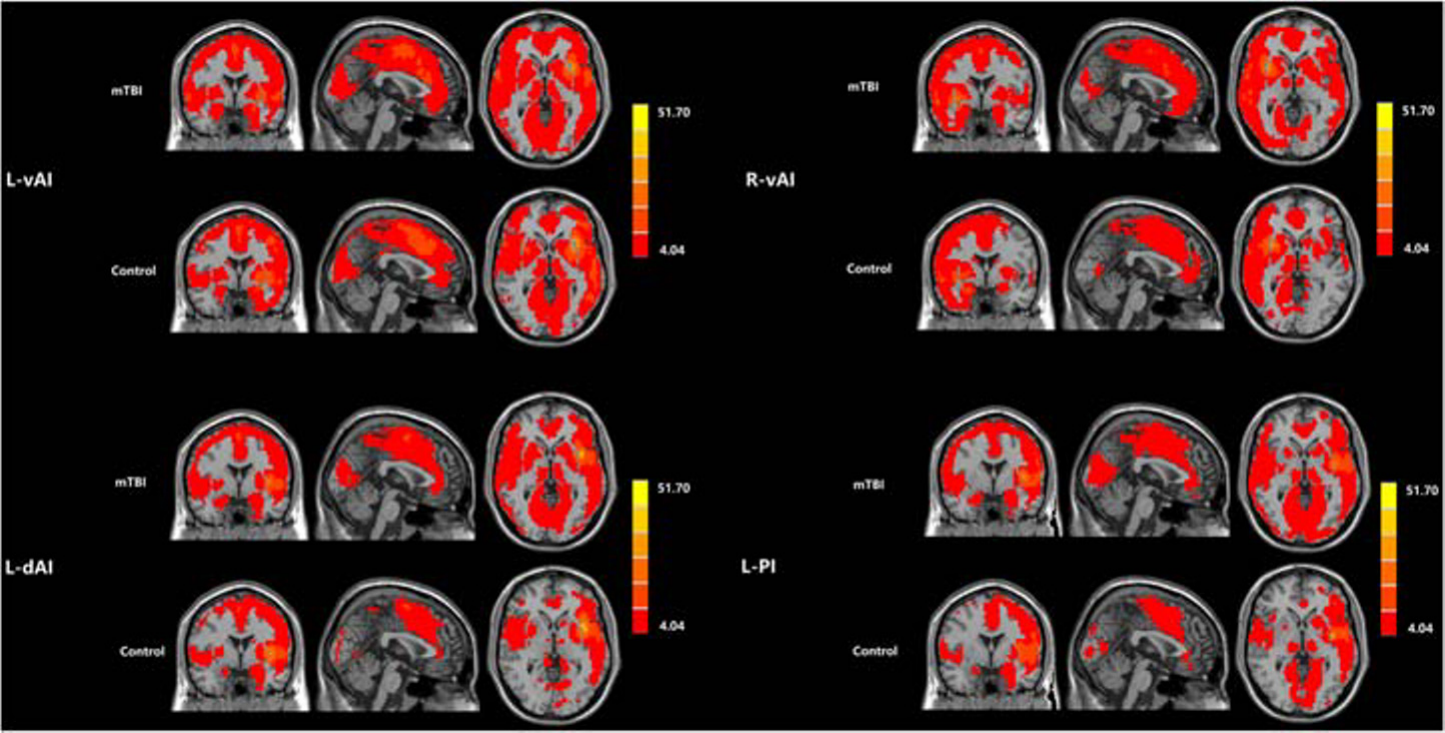
The functional connectivity maps of four insula seed ROIs (L-vAI, R-vAI, L-dAI, L-PI) were revealed in both mTBI patients and healthy controls

Figure 2 revealed the functional connectivity maps of four insula seed ROIs (L-vAI, R-vAI, L-dAI, L-PI) in both mTBI patients and healthy controls. The insula subdivisions mainly exhibited positive functional connectivity with the middle temporal gyrus, frontal, parietal and cingulate cortex.

**Fig. 3:**
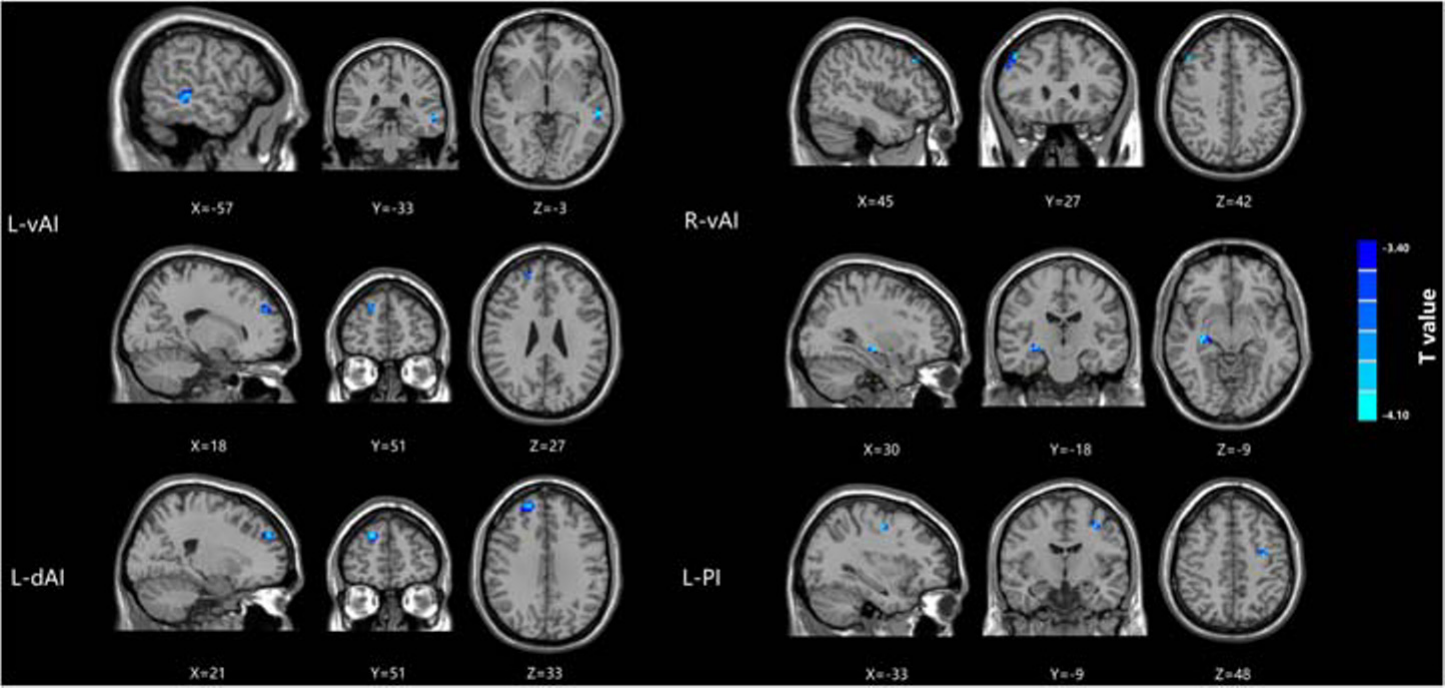
Significantly decreased functional connectivity of four insula seed ROIs (L-vAI, R-vAI, L-dAI, L-PI) in mTBI patients compared with healthy controls

Compared with healthy controls, patients with mTBI demonstrated significantly decreased functional connectivity between the L-vAI and the left middle temporal gyrus and right superior frontal gyrus and significantly decreased functional connectivity between the R-vAI and the right middle frontal gyrus and right hippocampus. While significantly decreased functional connectivity were observed between the L-dAI and the right superior frontal gyrus. Additionally, patients with mTBI demonstrated significantly decreased functional connectivity was observed between the L-PI and the left inferior frontal gyrus (Fig. 3; Table 2).

**Table 2:** Brain regions showing significant differences between mTBI and healthy controls

Brain region	BA	Peak MNI coordinates x,y,z(mm)	t value	Voxels
L-vAI				
L middle temporal gyrus	21	-57,-33,-3	-4.4124	24
R superior frontal gyrus	9	18,51,27	-3.9607	13
R-vAI				
R middle frontal gyrus	46	45,27,42	-4.0827	28
R hippocampus	35	30,-18,-9	-4.108	25
L-dAI				
R superior frontal gyrus	10	21,51,33	-5.2372	113
L-PI				
L middle frontal gyrus	46	-33,-9,48	-4.2604	13

A corrected threshold of *p* > determined by Monte Carlo simulation was taken as measuring that there was significant difference between groups. BA, Brodmann area; MNI, Montreal Neurological Institute; L, Left; R, Right

**Fig. 4:**
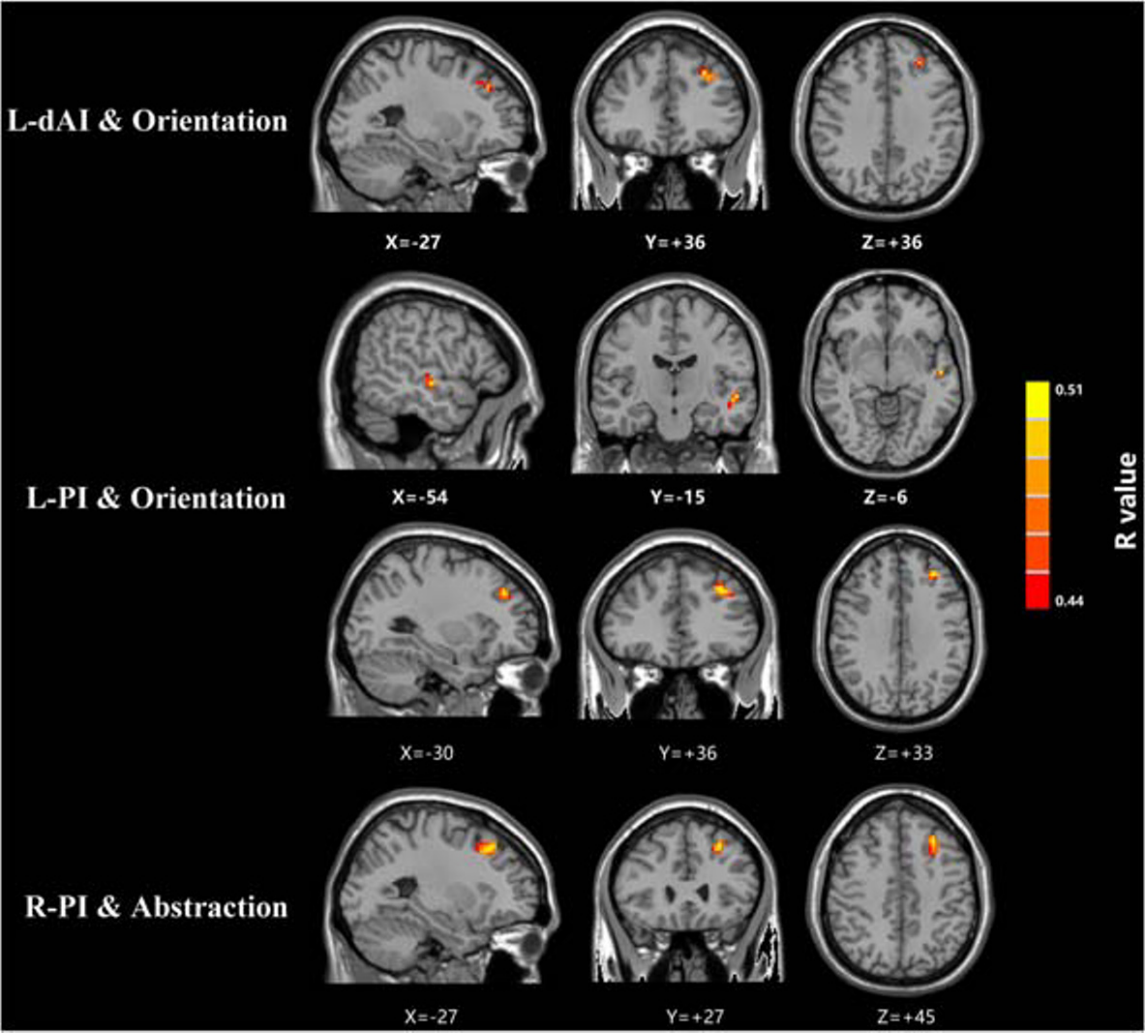
Voxel-wise correlations showed that the mTBI group demonstrated positive correlations between orientation performances and insular functional connectivity with middle temporal gyrus and superior and middle frontal gyrus. Abstraction scores in mTBI patients positively correlated with functional connectivity between the insula and middle frontal gyrus

As demonstrated in Fig. 4, the mTBI group demonstrated positive correlations between performances in orientation and insula functional connectivity with middle temporal gyrus and superior and middle frontal gyrus. Abstraction scores for mTBI patients positively correlated with functional connectivity between insula and middle frontal gyrus. The brain regions associated with MoCA sub-scores were described in Table 3.

**Table 3:** Brain regions associated with MoCA subscores in mTBI patients

Brain region	BA	Peak MNI coordinates x,y,z(mm)	Peak R value	Voxels
L-dAI and MoCA-Orientation				
L superior frontal gyrus	10	-27,36,36	0.50869	28
L-PI and MoCA-Orientation				
L middle temporal gyrus	21	-54,-15,-6	0.50849	15
L middle frontal gyrus	46	-30,36,33	0.52059	34
R-PI and MoCA-Abstraction				
L middle frontal gyrus	46	-27,27,45	0.54749	23

A corrected threshold of *p* > determined by Monte Carlo simulation was taken as measuring that there was significant difference between groups. BA, Brodmann area; MNI, Montreal Neurological Institute; L, Left; R, Right

## Discussion

We have previously observed that the functional connectivity of a.i. with other brain regions is impaired in mTBI patients during the acute stage (Li et al. 2019; Lu et al. 2019). However, the connectivity patterns of insula subdivisions to other brain regions within mTBI patients as compared to healthy controls have not been further studied. In this study, we not only found that insular sub-regions were abnormally connected with regions of the superior frontal gyrus, middle frontal gyrus, middle temporal gyrus and hippocampus but also found correlation between MoCA sub-scores and functional connectivity of insular subdivisions. These findings may shed new light to understand the pathophysiology of cognitive impairment following acute mTBI. Clinically, these features which show more accurate and comprehensive information may serve as objective biomarkers to achieve clinically-relevant capabilities for the diagnosis and treatment of mTBI, finally to improve the prognosis of mTBI.

## Insula-Hippocampal connectivity

In our study, the patients with mTBI presented decreased functional connectivity between the right vAI and right hippocampus. We also find in the literature examples where TBI patients exhibit smaller hippocampal volumes when compared to controls (Monti et al. 2013; Zhang et al. 2014; Sampedro et al. 2019). These results can be explained by the fact that hippocampus is believed disproportionately affected by mTBI because it is located in the medial temporal lobe of brain, which makes it more vulnerable to impact forces (Monti et al. 2013). Further, it is also particularly susceptible to excitotoxic injury, which often occurs in TBI. In addition to moderate and severe TBI, mTBI also could induce diffused hippocampal neuronal damages and apoptosis (Monti et al. 2013; Spielberg et al. 2015). And hippocampal neurodegeration in patients with mTBI linked to cognitive function decline is reported by previous studies (Monti et al. 2013; Zuo et al. 2018). However, the present analysis did not show any functional connectivity between the insula and hippocampus was significantly correlated with the MoCA score. Such inconsistency can be only partially explained by differences in the parameters used for image acquisition, the procedures used for data processing and analysis, and small sample sizes.

## Insula-Frontal connectivity

Compared to healthy controls, the current findings showed decreased functional connectivity between the insula and frontal gyrus in patients with mTBI, which is consistent with the fact that insula functionally connected with adjacent frontal regions. In many previous research, reduced neural activity in the inferior frontal gyrus, middle and superior frontal gyrus were found in mTBI (Iaria et al. 2008; Wang et al. 2015). One previous fMRI meta-analysis demonstrated a reason of a frontal vulnerability to mTBI, compared to controls (Eierud et al. 2014). The frontal lobes, which participate in frontal-subcortical circuits, play a critical role in cognitive function (Iaria et al. 2008). Meanwhile, our study demonstrated the functional connectivity between the insula and frontal gyrus had a significantly positive correlation with MoCA sub-scores including the orientation and abstraction scores. That is, the lower the functional connectivity, the lower the MoCA score. The mTBI possibly have damage on the frontal-subcortical circuits or white matter tracts, thereby inducing cognitive impairment. Compared with other domains of MoCA, orientation and abstraction are the cognitive processes found often to be affected after injury. Furthermore, abstract thinking was the most affected and showed minimal improvement at the time of discharge, but orientation showed maximum improvement. In fact, the parietal gyrus mainly controls the orientation function, and the frontal gyrus functionally and structurally connects with the parietal gyrus. Therefore, frontal gyrus affects the orientation and abstraction scores may via the relationship with the parietal gyrus. More related studies must be performed in the future.

## Insula-Temporal connectivity

In our data, the insula was found to be negatively connected with temporal gyrus, which matches the fact of a strong projection between the insula and temporal gyrus. Previous literature reported volume loss of gray matter in the temporal gyrus in mTBI (Babcock et al. 2015; Wang et al. 2019). Unsurprisingly, we observed decreased functional connectivity between the insula and temporal gyrus in patients with mTBI, compared with control group. As noted above, the frontal gyrus and the temporal gyrus play an important role in a variety of cognitive functions. These brain areas are also known to be the site of the pathophysiological foundations of cognitive impairment caused by the early stages of the disease. Moreover, previous literature found notable accumulations of amyloid in the temporal gyrus in patients who sufferer with significant cognitive dysfunction which suggests partial reason why temporal gyrus is related to cognitive performance (Mohamed et al. 2018). In addition, our study showed positive correlations between functional connectivity of insula and temporal gyrus and orientation score. In fact, orientation function is mainly charged by the parietal gyrus, the relationship between the insula or the temporal gyrus and orientation function is unclear. We presumed that the orientation function was affected by the insula or the temporal gyrus via the parietal gyrus because the posterior superior part of temporal gyrus is connected with parietal gyrus. However, the relationship between the temporal gyrus and parietal gyrus in acute mTBI patients must be further investigated.

## Limitations

There existed a number of limitations in our study. First, this study is limited to homogeneity in research populations of mTBI patients with different injury mechanisms and various brain injury sites. Second, GCS score was used to identify the severity of head injury. However, duration of loss of consciousness as an injury index is not considerable for most mTBI patients. Finally, this study only investigated functional connectivity dysfunction of insula subdivisions with other cortical regions at the acute stage, sub-acute and chronic data are necessary to improve to understand the development of damage and recovery. It may be important in future work.

## Conclusions

Taken together, our current and past work suggest that patients with acute mTBI suffer from functional connectivity dysfunction of insula subdivisions with other cortical regions including the hippocampus, frontal gyrus and temporal gyrus in comparison with healthy controls. Additionally, these patients showed significantly positive correlations between function connectivity of insula with above regions and cognitive performance.
